# Chicken blastoderms and primordial germ cells possess a higher expression of DNA repair genes and lower expression of apoptosis genes to preserve their genome stability

**DOI:** 10.1038/s41598-021-04417-y

**Published:** 2022-01-07

**Authors:** Deivendran Rengaraj, Sohyoung Won, Kyung Min Jung, Seung Je Woo, Haerang Lee, Young Min Kim, Heebal Kim, Jae Yong Han

**Affiliations:** 1 Department of Agricultural Biotechnology, and Research Institute of Agriculture and Life Sciences, Seoul National University, Seoul, 08826 Korea; 2 Interdisciplinary Program in Bioinformatics, Seoul National University, Seoul, 08826 Korea; 3 C&K Genomics, Seoul, 05836 Korea

**Keywords:** Cell biology, Computational biology and bioinformatics, Developmental biology, Genetics

## Abstract

DNA is susceptible to damage by various sources. When the DNA is damaged, the cell repairs the damage through an appropriate DNA repair pathway. When the cell fails to repair DNA damage, apoptosis is initiated. Although several genes are involved in five major DNA repair pathways and two major apoptosis pathways, a comprehensive understanding of those gene expression is not well-understood in chicken tissues. We performed whole-transcriptome sequencing (WTS) analysis in the chicken embryonic fibroblasts (CEFs), stage X blastoderms, and primordial germ cells (PGCs) to uncover this deficiency. Stage X blastoderms mostly consist of undifferentiated progenitor (pluripotent) cells that have the potency to differentiate into all cell types. PGCs are also undifferentiated progenitor cells that later differentiate into male and female germ cells. CEFs are differentiated and abundant somatic cells. Through WTS analysis, we identified that the DNA repair pathway genes were expressed more highly in blastoderms and high in PGCs than CEFs. Besides, the apoptosis pathway genes were expressed low in blastoderms and PGCs than CEFs. We have also examined the WTS-based expression profiling of candidate pluripotency regulating genes due to the conserved properties of blastoderms and PGCs. In the results, a limited number of pluripotency genes, especially the core transcriptional network, were detected higher in both blastoderms and PGCs than CEFs. Next, we treated the CEFs, blastoderm cells, and PGCs with hydrogen peroxide (H_2_O_2_) for 1 h to induce DNA damage. Then, the H_2_O_2_ treated cells were incubated in fresh media for 3–12 h to observe DNA repair. Subsequent analyses in treated cells found that blastoderm cells and PGCs were more likely to undergo apoptosis along with the loss of pluripotency and less likely to undergo DNA repair, contrasting with CEFs. These properties of blastoderms and PGCs should be necessary to preserve genome stability during the development of early embryos and germ cells, respectively.

## Introduction

Embryonic development in chicken is classified into stages by two major criteria: Eyal-Giladi and Kochav (EGK) criteria^1^ that classify the intrauterine embryonic development; and Hamburger and Hamilton (HH) criteria^2^ that classify the post-ovipositional development. At EGK stage X (EGK.X) blastoderm, the area pellucida and area opaca regions are clearly distinguishable. The area pellucida consists of 1–2 cell layers with an upper continuous single-cell layered epiblast and a lower discontinuous hypoblast layer, while the area opaca is multilayered^3,4^. The EGK.X blastoderm is composed of mostly undifferentiated progenitor (pluripotent) cells with the potency to differentiate into all cell types, and blastoderm-derived chicken embryonic stem cells (ESCs) have been established in vitro^5,6^. Primordial germ cells (PGCs) are also undifferentiated progenitor cells found initially in the blastoderm and have the potency to differentiate into germline cells^7^. In chickens, PGCs undergo dynamic migration and differentiation processes in the early embryos. PGCs originate in the area pellucida of intrauterine embryos and eventually migrate to the germinal crescent during the formation of primitive streak^8,9^. After forming the extraembryonic blood vessels, PGCs migrate from the germinal crescent to the bloodstream and circulates in the blood until the formation of embryonic gonads^7^. In embryonic gonads, PGCs undergo rapid proliferation and subsequently differentiate into male germ cells (that further enter into mitotic arrest) or female germ cells (that further enter into meiotic arrest)^10^.

Analyzing the transcriptional programs of blastoderms is critical to understanding the molecular mechanisms that underlie the developmental fate of early embryos and the practical application of blastoderm-derived pluripotent cells, including ESCs. Similarly, analyzing the transcriptional programs of PGCs is critical to understanding the molecular mechanisms that underlie the developmental fate of early germ cells and the practical application of PGCs in generating transgenic chickens. Fibroblasts are abundant somatic cells found in many tissues and have several advantages in biological sciences. Fibroblasts play critical roles in regulating extracellular matrices, interstitial fluid volume and pressure, and wound healing^11^. The chicken embryonic fibroblasts (CEFs) were successfully used for the amplification of viruses. However, the primary cultured cells have a limited life span^12,13^. We have routinely used CEFs as one of the reference samples to compare the expression and functions of genes in blastoderms/ESCs/PGCs^14–16^. In the current study, we performed whole-transcriptome sequencing (WTS)-based expression profiling of DNA repair pathway, apoptosis pathway, and pluripotency regulating genes in EGK.X blastoderms and PGCs (target samples) compared with CEFs (reference sample).

DNA is the fundamental unit of inheritance and is susceptible to damage by various exogenous and endogenous sources. The exogenous sources include, for instance, ultraviolet (UV) radiation, ionizing radiation (X-ray and gamma rays), incorporated radioisotopes, and a variety of genotoxic chemicals (vinyl chloride, hydrogen peroxide, alkylating agents, aromatic amines, polycyclic aromatic hydrocarbons), while the endogenous sources mainly include, intracellular oxidative stress and errors in DNA replication^17–19^. Thus, genome integrity in cells is constantly monitored by a complex and highly conserved cellular mechanism, collectively termed the DNA damage response (DDR), which senses the DNA damage, arranges the appropriate repair of DNA damage, and resolve the DNA replication problems^20,21^. There are five major DNA repair pathways that the cell uses to repair the DNA damage. The pathways include base excision repair (BER), nucleotide excision repair (NER), mismatch repair (MMR), non-homologous end joining (NHEJ), and homologous recombination (HR). Of these pathways: BER, NER, and MMR repairs single-strand break; BER and NER repairs base damage; NHEJ repairs double-strand break, however, with errors; HR repairs double-strand break error-free; and all pathway correct non-double-strand break clustered DNA lesions^18,19,22^. These pathways are critical for maintaining the genome stability of cells. When DNA repair fails in cells, cell cycle progression is halted, and apoptosis is initiated. Also, incomplete or misrepair can result in a progeny of cells with mutations and chromosomal instability^18^. Apoptosis occurs through two main pathways: the extrinsic pathway, activated by ligand binding of a subset of the TNF receptor superfamily at the cell membrane; and the intrinsic pathway, controlled by the BCL-2 protein family and executed by mitochondrial outer membrane permeabilization (MOMP). Both pathways lead to the final activation of caspase-3 and caspase-7, which cleave over 1000 proteins leading to their degradation or activation^23^.

The genome integrity of pluripotent stem cells and germ cells is essential to the permanence of all species. DNA-damaged cells delay cell-cycle progression through checkpoint control and DNA repair for genome stability, but in some cases, directly induce apoptosis to eliminate mutated cells^24,25^. In particular, in the case of ESCs and induced pluripotent stem cells (iPSCs), apoptosis sensitivity is conspicuously conserved in single-strand and double-strand DNA breakage^26–28^. The genome integrity in human ESCs was controlled by removing damaged cells through apoptosis mechanism rather than DNA repair when exposed to DNA replication inhibitors^26^. Also, human ESCs undergo more rapid p53-dependent apoptosis after DNA damage than differentiated cells^29^. Although the germ cell also has high apoptotic potential, it is known that apoptosis is well induced when genetic mutations occur^30^. In a study of medaka fish using p53 and msh2 deficient germ cells, it was found that p53 and msh2 genes inhibit radiation-induced microsatellite instability (MSI) through mismatch removal and apoptosis mechanisms^30^. Thus, pluripotent cell types have different DNA damage and apoptosis mechanisms than differentiated cell types, which may control genetic instability against defects in their genome or epigenome.

A comprehensive understanding of the conservation and expression of DNA repair and apoptosis pathway genes is not very clear in chicken tissues. Therefore, our report on the expression profiling of DNA repair and apoptosis pathway genes along with pluripotency regulating genes in CEFs, EGK.X blastoderms, and PGCs at normal- and DNA damage-induced conditions will provide insights into the genome stability and instability of these test samples.

## Results

### Whole-transcriptome sequencing of CEFs, EGK.X blastoderms, and PGCs

**Figure 1:**
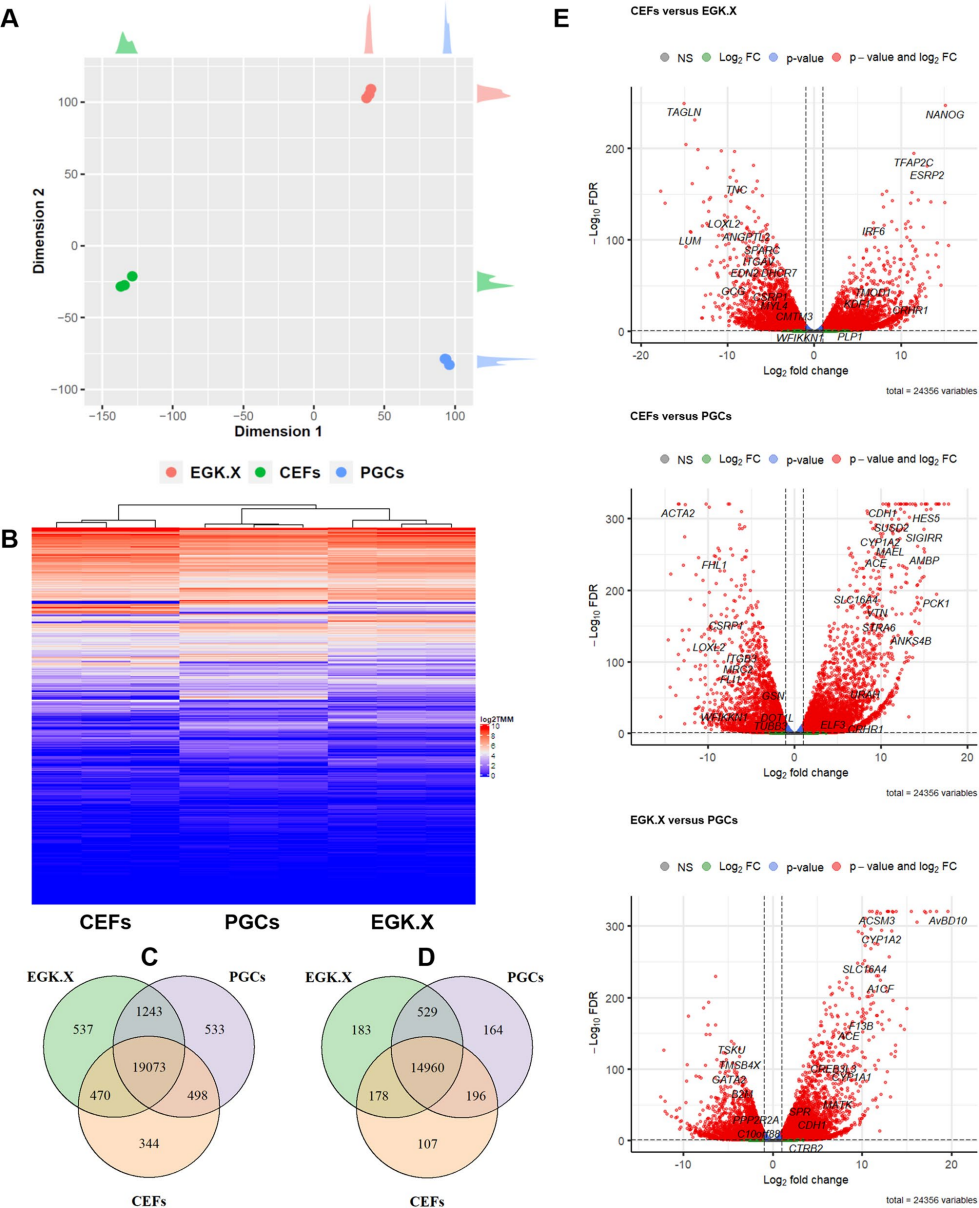
Investigation of the relationships between CEFs, EGK.X blastoderms, and PGCs based on quantified genes (24,356) expression of the whole-transcriptome sequencing. (**A**) MDS plot approach. (**B**) Heatmap approach. (**C**) Venn diagram approach using all types of genes. (**D**) Venn diagram approach using protein-coding genes. (**E**) Volcano plot approach. In (**E**), three statistical tests were performed between the test samples: CEFs versus EGK.X blastoderms; CEFs versus PGCs; and EGK.X blastoderms versus PGCs. Significantly upregulated (right) and downregulated (left) genes in EGK.X blastoderms compared with CEFs (top graph), PGCs compared with CEFs (middle graph), and PGCs compared with EGK.X blastoderms (bottom graph).

The total RNA samples of CEFs, EGK.X blastoderms, and PGCs were used to construct cDNA libraries. The resulting libraries were subjected to WTS analysis using the Illumina Nextseq 500 platform. The raw sequencing data were deposited in the NCBI GEO database (GSE174603). On average, among the Illumina Nextseq 500 platform produced raw-paired end reads, 126,023,088 reads (93.91%) were QC passed clean reads. Of the QC passed reads, 108,639,238 paired-end reads (86.46%) were mapped to the chicken reference genome (galGal6a) (Table S1), and those reads were mapped to 24,356 genes after filtering out non-mapped genes across all samples. The summary of transcript types detected in CEFs, EGK.X blastoderms, and PGCs are shown in Table S2. Based on the total quantified genes (24,356), the relationships between CEFs, EGK.X blastoderms, and PGCs were investigated through several approaches. According to MDS plot approach, CEFs, EGK.X blastoderms, and PGCs samples were clearly divergent (Fig. 1A). According to heatmap and Venn diagram approaches, transcripts commonly or uniquely expressed in the test samples were clearly revealed (Fig. 1B–D). In particular, among all types of genes, 344, 537, and 533 were uniquely expressed in CEFs, EGK.X blastoderms, and PGCs, respectively (Fig. 1C). Among the protein-coding genes, 107, 183, and 164 were uniquely expressed in CEFs, EGK.X blastoderms, and PGCs, respectively (Fig. 1D). Statistical tests were performed to identify differentially expressed genes (DEGs) between the test samples: i.e., CEFs versus EGK.X blastoderms; CEFs versus PGCs; and EGK.X blastoderms versus PGCs. As a result, large numbers of DEGs were detected in EGK.X blastoderms/PGCs compared with CEFs at FDR-adjusted *P* < 0.05. Similarly, large numbers of DEGs were detected in PGCs compared with EGK.X blastoderms at FDR-adjusted *P* < 0.05 (Fig. 1E).

### Analysis of the relationship between DNA repair pathway, apoptosis pathway, and pluripotency regulating genes

The chicken-specific DNA repair pathway genes were primarily identified using the Kyoto Encyclopedia of Genes and Genomes (KEGG) pathway maps. The presence of those identified genes in the current annotation release of the chicken reference genome (galGal6a) was further verified using the Ensembl database. As a result, 27 BER pathway genes, 38 NER pathway genes, 19 MMR pathway genes, 10 NHEJ pathway genes, and 39 HR pathway genes were identified (Table S3). The identified gene list of each DNA repair pathway was again subjected to the KEGG mapper. The results revealed that the DNA repair pathway genes are also involved in several other pathways related to, for instance, cell communication, cell signaling, cell cycle, and DNA replication. Furthermore, several genes were found to have a versatile role in DNA repair as they were overlapped between the DNA repair pathways (Fig. S1). Similarly, we identified the chicken-specific apoptosis (including extrinsic and intrinsic) pathway genes using the KEGG pathway map and verified their presence in the galGal6a using the Ensembl database. The identified 22 apoptosis pathway genes (Table S3) were again subjected to KEGG pathway mapper. The results revealed that they are involved in more other pathways (Fig. S1) than DNA repair pathway genes. Since the chicken-specific information is not available, the signaling pathway genes regulating pluripotency of stem cells in human/mouse were retrieved using the KEGG pathway map, and their homologs in chicken were identified using the Ensembl database. In total, 134 homologs of the pluripotency regulating genes were identified in chicken; however, none of them were overlapped with the DNA repair pathway or apoptosis pathway genes. For further analysis, we selected 25 candidate pluripotency regulating genes (Table S3) that represent different signaling pathways (Jak-STAT, MAPK, TGFβ, Wnt, and PI3K-AKT), core transcriptional network, and downstream targets (essential for pluripotency maintenance).

We clustered the protein-coding genes (16,317 genes whose expression is not zero), including the DNA repair, apoptosis, and pluripotency genes (shown in Table S3), into 100 clusters. As a result, 22 clusters contain only DNA repair pathway genes, 6 clusters contain apoptosis pathway genes, and 11 clusters contain pluripotency regulating genes, indicating their unique expression patterns and role in these activities (Table S4). In particular, at least 8 clusters (clusters 6, 9, 20, 23, 25, 30, 49, and 74) contain only three or more DNA repair pathway genes, further indicating subsets of the DNA repair pathway genes showing similar expression patterns. In the case of clusters containing pluripotency regulating core transcriptional network genes, *NANOG* and *Pou5f3* were present within cluster 99, but *SOX2* was present in cluster 29, indicating the distinct expression patterns of *SOX2*. Furthermore, at least 4 clusters (clusters 2, 10, 31, and 43) contain genes from all three categories (DNA repair pathway, apoptosis pathway, and pluripotency regulating). Other clusters contain genes from any two of the three categories (DNA repair and apoptosis, DNA repair and pluripotency, or apoptosis and pluripotency). Here, we can infer that a number of DNA repair, apoptosis, and pluripotency genes show related expression and connected functions, possibly through signaling genes.

### Expression profiling of DNA repair pathway genes in CEFs, EGK.X blastoderms, and PGCs

**Figure 2:**
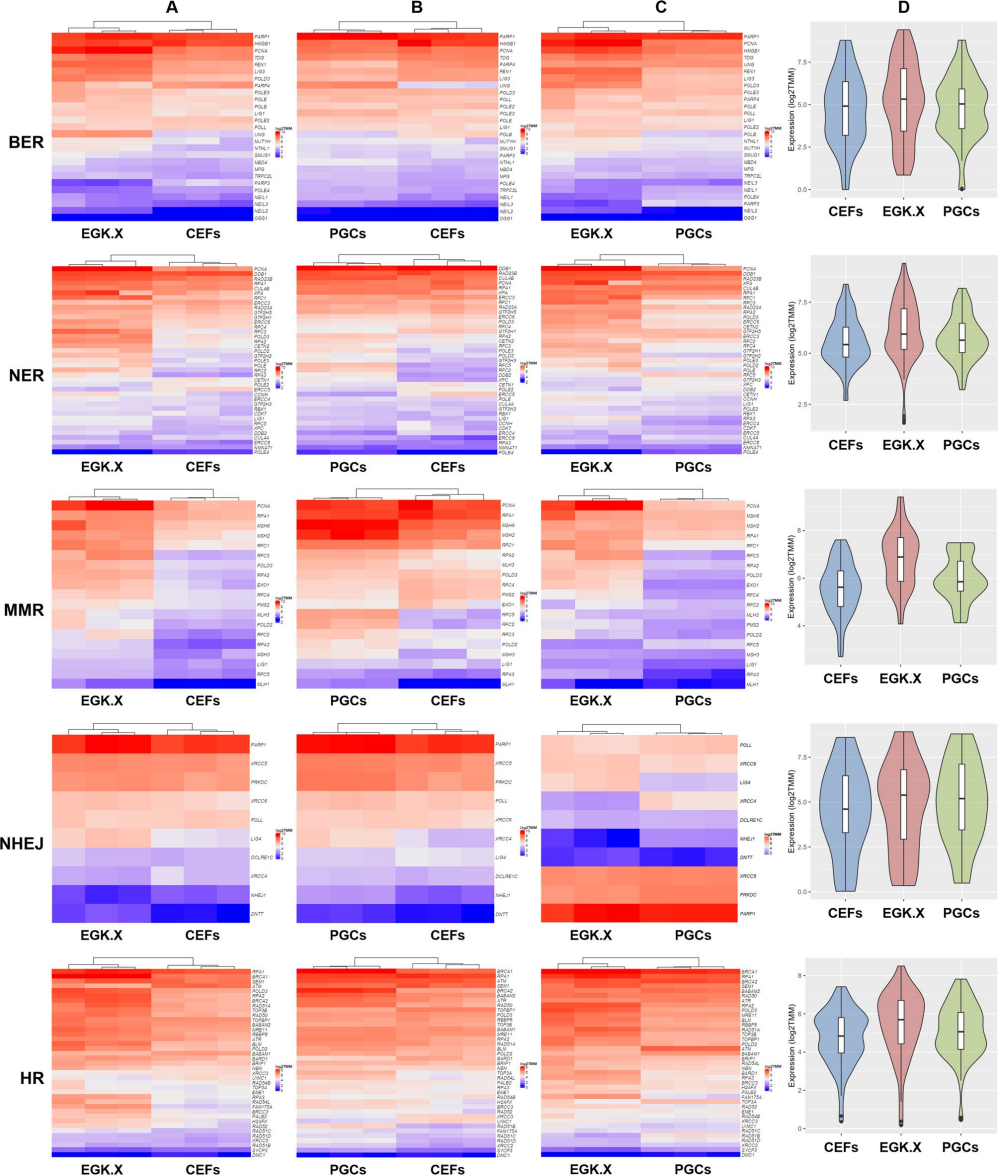
Visualization of the expression of DNA repair pathway genes. (**A–C**) Heatmaps using log_2_ *TMM* values of the normalized read counts of each DNA repair pathway genes in CEFs and EGK.X blastoderms (**A**), CEFs and PGCs (**B**), and EGK.X blastoderms and PGCs (**C**) were prepared separately for better visualization. (**D**) Violin plots were prepared with the log_2_ *TMM* values to visualize the overall expression of DNA repair pathway genes in CEFs, EGK.X blastoderms, and PGCs.

**Figure 3:**
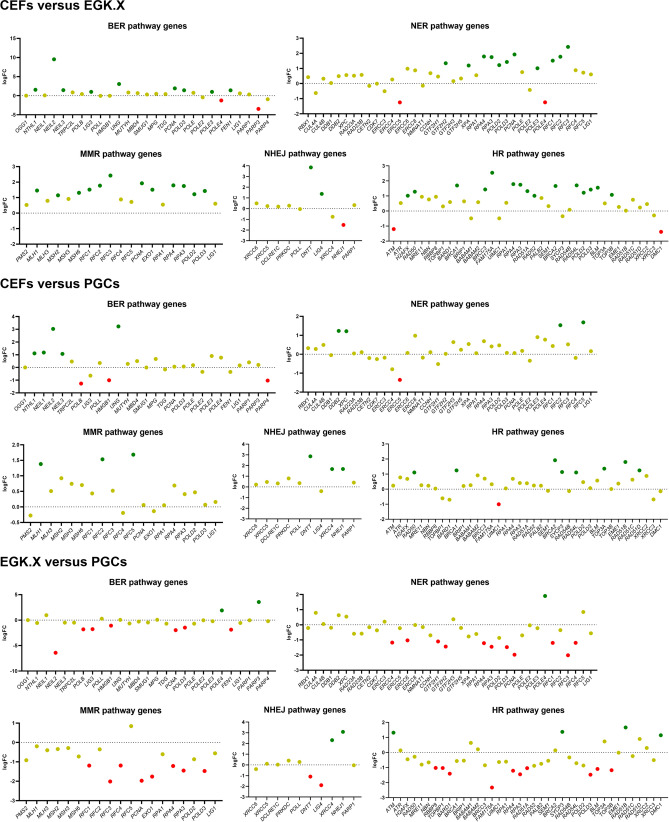
Differentially expressed genes (DEGs) of the DNA repair pathway between the test samples. DEGs in EGK.X blastoderms compared with CEFs, DEGs in PGCs compared with CEFs, and DEGs in PGCs compared with EGK.X blastoderms were shown in the top, middle, and bottom graphs, respectively. Green: significantly upregulated. Red: significantly downregulated. Yellow: unregulated.

We screened the read counts of DNA repair pathway genes based on the WTS of CEFs, EGK.X blastoderms, and PGCs. Then, we used the log_2_ *TMM* values of the normalized read counts for better visualization of the gene expression through heatmaps. In the results, most of the DNA repair pathway genes were expressed stronger in EGK.X blastoderms than CEFs (Fig. 2A). Also, most of the DNA repair pathway genes were expressed stronger in PGCs than CEFs (Fig. 2B). Moreover, when we compared EGK.X blastoderms and PGCs, the DNA repair pathway genes were expressed stronger in EGK.X blastoderms than PGCs (Fig. 2C). The overall expression of DNA repair pathway genes in CEFs, EGK.X blastoderms, and PGCs were visualized through violin plots with the log_2_ *TMM* values of genes. As a result, the overall expression of DNA repair pathway genes was stronger in EGK.X blastoderms (Fig. 2D). Next, DEGs of the DNA repair pathway were examined between the test samples: CEFs versus EGK.X blastoderms; CEFs versus PGCs; and EGK.X blastoderms versus PGCs. According to FDR-adjusted *P* < 0.05 and logFC cutoff, 9 BER genes, 11 NER genes, 12 MMR genes, 2 NHEJ genes, and 15 HR genes were shown > 1-fold upregulation in EGK.X blastoderms than CEFs. In PGCs, 5 BER genes, 4 NER genes, 3 MMR genes, 3 NHEJ genes, and 8 HR genes were shown > 1-fold upregulation than CEFs. Only 1–3 genes of some DNA repair pathways were shown < − 1-fold downregulation in EGK.X blastoderms/PGCs than CEFs. In EGK.X blastoderms versus PGCs condition, 7 BER genes, 11 NER genes, 8 MMR genes, 2 NHEJ genes, and 10 HR genes were shown < − 1-fold downregulation in PGCs (i.e., they are > 1-fold upregulated in EGK.X blastoderms) (Fig. 3). Looking at the results of gene set enrichment analysis (GSEA) (Fig. S2), the DNA repair pathways were shown enrichment in EGK.X blastoderms (Fig. S2A), and PGCs (Fig. S2B) compared to CEFs as more genes (black bars) are in the positively correlated region. Also, the DNA repair pathways were shown enrichment in EGK.X blastoderms compared to PGCs as more genes are in the negatively correlated region (Fig. S2C). Although the GSEA results were not significant at the confidence level of 0.05, we can find a similar tendency as in Figs. 2 and 3.

We employed quantitative reverse transcription-polymerase chain reaction (RT-qPCR) to further verify a part of WTS results. We selected candidate genes from the BER pathway (*NEIL2* and *UNG*), NER/MMR pathway (*PCNA* and *RFC3*), NHEJ pathway (*DNTT* and *LIG4*), and HR pathway (*BRCA1* and *FAM175A*), which showed higher fold expression primarily in EGK.X blastoderms in the WTS results. The selected genes were amplified using the gene-specific primers (Table S5) and prepared cDNA of test samples. All the selected genes were significantly expressed more higher in EGK.X blastoderms than their expression in CEFs (Fig. S3), consistent with WTS results. Also consistent with WTS results, *NEIL2*, *UNG*, *DNTT*, and *BRCA1* were significantly expressed higher in PGCs compared to CEFs expression (Fig. S3).

### Expression profiling of apoptosis pathway genes in CEFs, EGK.X blastoderms, and PGCs

**Figure 4:**
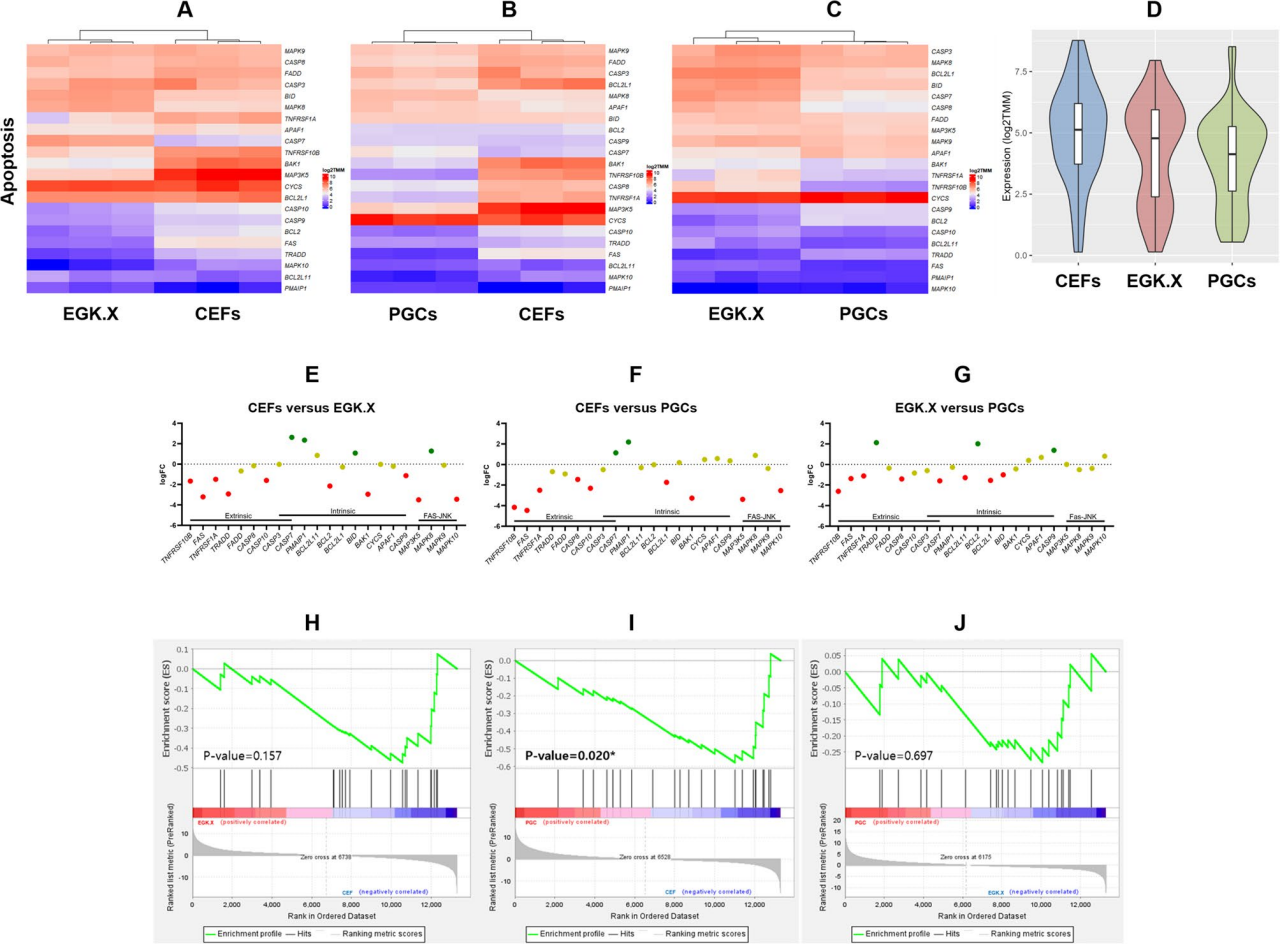
Expression profiling of apoptosis pathway genes. (**A–C**) Heatmaps using log_2_ *TMM* values of the normalized read counts of apoptosis pathway genes in CEFs and EGK.X blastoderms (**A**), CEFs and PGCs (**B**), and EGK.X blastoderms and PGCs (**C**) were prepared separately for better visualization. (**D**) Violin plots were prepared with the log_2_ *TMM* values to visualize the overall expression of apoptosis pathway genes in CEFs, EGK.X blastoderms, and PGCs. (**E–G**) DEGs of apoptosis pathway between the test samples. DEGs in EGK.X blastoderms compared with CEFs, DEGs in PGCs compared with CEFs, and DEGs in PGCs compared with EGK.X blastoderms were shown in (**E**–**G**) respectively. Green: significantly upregulated. Red: significantly downregulated. Yellow: unregulated. (**H–J**) GSEA of apoptosis pathway at CEFs versus EGK.X blastoderms (**H**), CEFs versus PGCs (**I**), and EGK.X blastoderms versus PGCs (**J**) conditions.

According to log_2_ *TMM* values of the normalized read counts of apoptosis pathway genes based on the WTS of CEFs, EGK.X blastoderms, and PGCs, most of the apoptosis pathway genes were expressed lower in EGK.X blastoderms (Fig. 4A) and PGCs (Fig. 4B) than that of CEFs. On the other hand, the expression of apoptosis pathway genes was stronger in EGK.X blastoderms than PGCs (Fig. 4C). The overall expression of apoptosis pathway genes was higher in CEFs (Fig. 4D). Next, DEGs of apoptosis pathway were examined between the test samples: CEFs versus EGK.X blastoderms; CEFs versus PGCs; and EGK.X blastoderms versus PGCs. According to FDR-adjusted *P* < 0.05 and logFC cutoff, 10 and 9 genes were shown < − 1-fold downregulation in EGK.X blastoderms (Fig. 4E) and PGCs (Fig. 4F), respectively, than CEFs. In EGK.X blastoderms versus PGCs condition, 8 genes were shown < − 1-fold downregulation in PGCs (i.e., they are > 1-fold upregulated in EGK.X blastoderms) (Fig. 4G). The GSEA results of the apoptotic pathway showed that the pathway genes appear to be expressed less in EGK.X blastoderms (Fig. 4H) and PGCs (Fig. 4I) than that of CEFs, and less in PGCs than that of EGK.X blastoderms (Fig. 4J), a similar tendency as in Fig. 4A–G.

To further confirm WTS results, we selected 4 candidate apoptosis genes (*FAS*, *TNFRSF1A*, *BAK1,* and *MAP3K5*) that showed higher fold downregulation in EGK.X blastoderms and PGCs. Then, we amplified them using the gene-specific primers (Table S5) and cDNA of test samples by RT-qPCR. Consistent with WTS results, *FAS*, *TNFRSF1A*, *BAK1,* and *MAP3K5* were expressed lower in EGK.X blastoderms and more lower in PGCs compared to CEFs expression (Fig. S3).

### Expression profiling of pluripotency regulating genes in CEFs, EGK.X blastoderms, and PGCs

**Figure 5:**
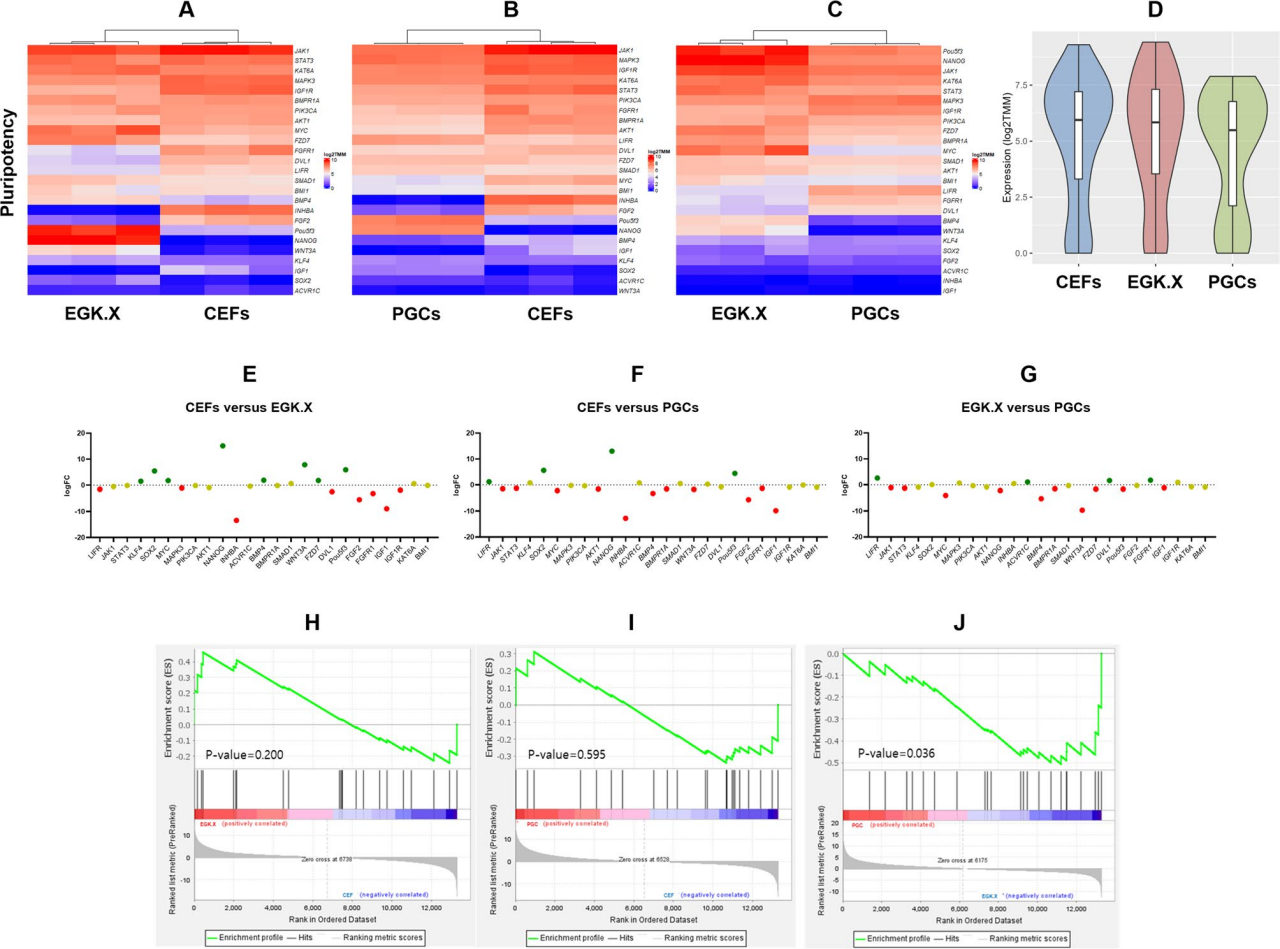
Expression profiling of pluripotency regulating genes. (**A–C**) Heatmaps using log_2_ *TMM* values of the normalized read counts of pluripotency regulating genes in CEFs and EGK.X blastoderms (**A**), CEFs and PGCs (**B**), and EGK.X blastoderms and PGCs (**C**) were prepared separately for better visualization. (**D**) Violin plots were prepared with the log_2_ *TMM* values to visualize the overall expression of pluripotency regulating genes in CEFs, EGK.X blastoderms, and PGCs. (**E–G**) DEGs of pluripotency regulating pathway between the test samples. DEGs in EGK.X blastoderms compared with CEFs, DEGs in PGCs compared with CEFs, and DEGs in PGCs compared with EGK.X blastoderms were shown in (**E**–**G**), respectively. Green: significantly upregulated. Red: significantly downregulated. Yellow: unregulated. (**H–J**) GSEA of pluripotency regulating genes at CEFs versus EGK.X blastoderms (**H**), CEFs versus PGCs (**I**), and EGK.X blastoderms versus PGCs (**J**) conditions.

We examined the expression profiling of 25 candidate pluripotency regulating genes in CEFs, EGK.X blastoderms, and PGCs based on the WTS data. According to log_2_ *TMM* values of the normalized read counts, and FDR-adjusted *P* < 0.05 and logFC cutoff, 8 genes each were shown > 1-fold upregulation or < − 1-fold downregulation in EGK.X blastoderms than CEFs. In particular, core transcriptional network genes (*NANOG*, *Pou5f3*, and *SOX2*), pluripotency-associated genes (*KLF4* and *MYC*), BMP signaling gene (*BMP4*), and Wnt signaling genes (*WNT3A* and *FZD7*) were upregulated in EGK.X blastoderms than CEFs (Fig. 5A,E). According to log_2_ *TMM* values of the normalized read counts, and FDR-adjusted *P* < 0.05 and logFC cutoff, only 4 genes were shown upregulation, but 11 genes were shown downregulation in PGCs compared to CEFs. The PGCs upregulated genes include *NANOG*, *Pou5f3*, *SOX2*, and *LIFR* (Fig. 5B,F). When we compared EGK.X blastoderms and PGCs, most of the pluripotency regulating genes were downregulated in PGCs than EGK.X blastoderms (Fig. 5C,G). Moreover, the overall expression of pluripotency regulating genes was higher in EGK.X blastoderms (Fig. 5D). The GSEA results of the pluripotency regulating genes further confirmed the positively correlated genes and negatively correlated genes in the test samples (Fig. 5H–J), a similar tendency as in Fig. 5A–G. Most of the significantly upregulated genes in EGK.X blastoderms and/or PGCs were reported in several earlier studies^16,31–34^. Therefore, we have not further confirmed these genes in this study.

### Cell status of CEFs, EGK.X blastoderm cells, and PGCs at DNA damage-induced condition

**Figure 6:**
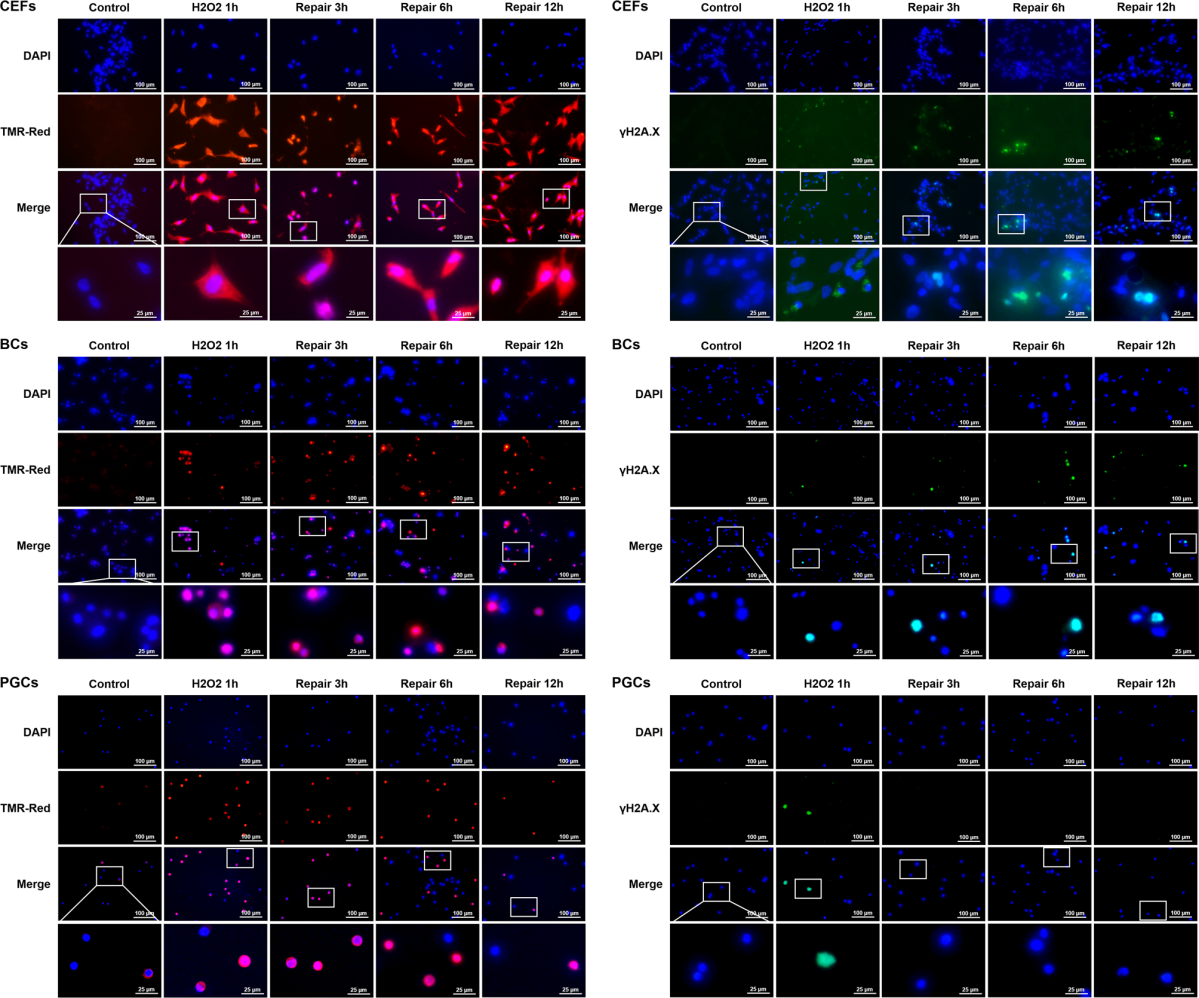
TUNEL assay and γ-H2A.X immunocytochemistry in CEFs, EGK.X blastoderm cells (BCs), and PGCs treated with H_2_O_2_. The cultured CEFs, EGK.X blastoderm cells, and PGCs were treated with 1 mM H_2_O_2_ for 1 h (H_2_O_2_ 1 h) in one group. In three other groups, the cells were treated with 1 mM H_2_O_2_ for 1 h, and then incubated in fresh media without H_2_O_2_ for 3 h (repair 3 h), 6 h (repair 6 h) and 12 h (repair 12 h). After treatment, cells were separately subjected for TUNEL assay with TMR-red staining (left panel) and γ-H2A.X immunocytochemistry (right panel).

The CEFs, EGK.X blastoderm cells, and PGCs were cultured in vitro, and one group of cells were treated with 1 mM H_2_O_2_ for 1 h (termed as H_2_O_2_ 1 h) to induce DNA damage. To know whether the affected cells undergo DNA repair, another three groups of cells were first treated with 1 mM H_2_O_2_ for 1 h, and then incubated in fresh media without H_2_O_2_ for 3 h (termed as repair 3 h), 6 h (repair 6 h) and 12 h (repair 12 h), respectively. Apoptotic cells with DNA strand breaks were identified using terminal deoxynucleotidyl transferase dUTP nick end labeling (TUNEL) assay and TMR-red staining. Cells undergoing DNA repair were identified using phosphorylated *H2A* histone family member *X* (γ-H2A.X) immunocytochemistry. As the results (Fig. 6), in CEFs, almost all of the cells were positive to TMR-red from H_2_O_2_ 1 h to repair 12 h groups, and a considerable number of cells were positive to γ-H2A.X from H_2_O_2_ 1 h to repair 12 h groups. In EGK.X blastoderm cells, many cells were positive to TMR-red from H_2_O_2_ 1 h to repair 12 h groups; however, only a few cells were positive to γ-H2A.X from H_2_O_2_ 1 h to repair 12 h groups. In PGCs, many cells were positive to TMR-red from H_2_O_2_ 1 h to repair 12 h groups; however, only a few cells were positive to γ-H2A.X, particularly at H_2_O_2_ 1 h group. Almost none of the PGCs were positive to γ-H2A.X from repair 3 h to repair 12 h groups.

**Figure 7:**
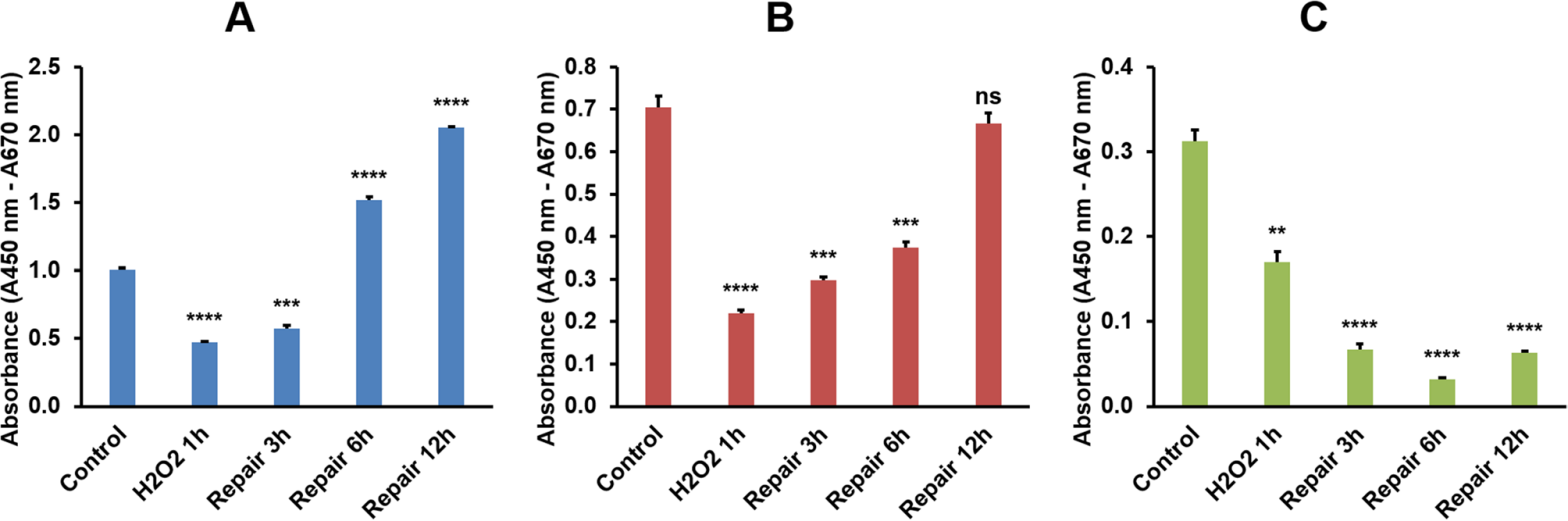
WST-1 assay in CEFs, EGK.X blastoderm cells (BCs), and PGCs treated with H_2_O_2_. The cultured CEFs (**A**), EGK.X blastoderm cells (**B**), and PGCs (**C**) were treated with 1 mM H_2_O_2_ for 1 h (H_2_O_2_ 1 h) in one group. In three other groups, the cells were treated with 1 mM H_2_O_2_ for 1 h, and then incubated in fresh media without H_2_O_2_ for 3 h (repair 3 h), 6 h (repair 6 h) and 12 h (repair 12 h). After treatment, cells were subjected for WST-1 assay. Significant differences between the respective control and treated samples were determined by Student’s *t*-test. Statistical significance was ranked as *P < 0.05, **P < 0.01, ***P < 0.001, or ****P < 0.0001. *ns*, non-significant.

We performed WST-1 assay to detect the relative proliferation rate of CEFs, EGK.X blastoderm cells, and PGCs in H_2_O_2_ 1 h to repair 12 h groups. Compared to the respective control, the proliferation rate of CEFs was significantly reduced at H_2_O_2_ 1 h and repair 3 h groups. The recovery of CEFs proliferation rate was significantly increased over control level at repair 6 h and 12 h groups (Fig. 7A). Compared to the respective control, the proliferation rate of EGK.X blastoderm cells was significantly reduced at H_2_O_2_ 1 h group. Though some level of recovered cell proliferation rate occurs during the repair time points, it does not reach the control level even at the repair 12 h group (Fig. 7B). Compared to the respective control, the proliferation rate of PGCs was detected significantly lower at all H_2_O_2_ 1 h to repair 12 h groups (Fig. 7C).

### Expression status of CEFs, EGK.X blastoderm cells, and PGCs at DNA damage-induced condition

**Figure 8:**
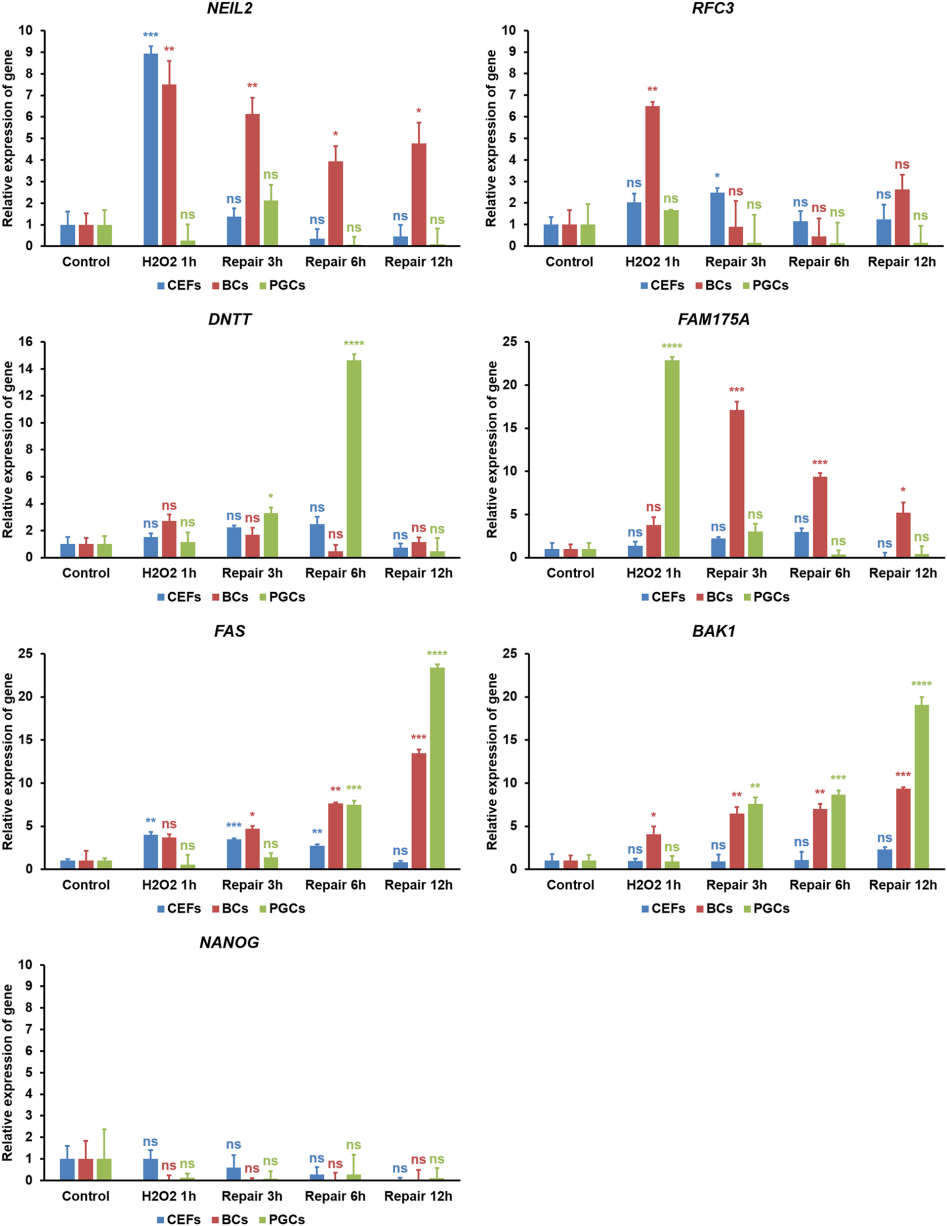
RT-qPCR analysis in CEFs, EGK.X blastoderm cells (BCs), and PGCs treated with H_2_O_2_. The cultured CEFs, EGK.X blastoderm cells, and PGCs were treated with 1 mM H_2_O_2_ for 1 h (H_2_O_2_ 1 h) in one group. In three other groups, the cells were treated with 1 mM H_2_O_2_ for 1 h, and then incubated in fresh media without H_2_O_2_ for 3 h (repair 3 h), 6 h (repair 6 h) and 12 h (repair 12 h). After treatment, cDNA of the cells was prepared and amplified with specific qPCR primers of the candidate genes from the BER pathway (*NEIL2*), NER/MMR pathway (*RFC3*), NHEJ pathway (*DNTT*), HR pathway (*FAM175A*), apoptosis pathway (*FAS* and *BAK1*), and pluripotency regulating pathway (*NANOG*). The relative expression of genes was normalized with the chicken *GAPDH* and respective control sample, and analyzed by the 2^−ΔΔCt^ method. Significant differences between the respective control and treated samples were determined by Student’s *t* test. Statistical significance was ranked as *P < 0.05, **P < 0.01, ***P < 0.001, or ****P < 0.0001. *ns*, non-significant.

We selected *NEIL2* from the BER pathway, *RFC3* from the NER/MMR pathway, *DNTT* from the NHEJ pathway, and *FAM175A* from the HR pathway. These genes were selected based on their higher fold expression primarily in EGK.X blastoderms and secondarily in PGCs at normal condition. We also selected *FAS* from the extrinsic apoptosis pathway and *BAK1* from the intrinsic apoptosis pathway, based on their higher fold downregulation in EGK.X blastoderms and PGCs. Among the pluripotency regulating genes, we selected *NANOG* that showed higher fold expression primarily in EGK.X blastoderms and secondarily in PGCs. The selected genes were amplified using the gene-specific primers (Table S5) and cDNA of CEFs, EGK.X blastoderm cells, and PGCs from H_2_O_2_ 1 h to repair 12 h groups. In the results of DNA repair pathway genes (Fig. 8), *NEIL2* expression was highest at H_2_O_2_ 1 h group in CEFs, significantly higher in a decreasing manner from H_2_O_2_ 1 h to repair 12 h groups in EGK.X blastoderm cells, and a slight increase at repair 3 h group in PGCs, compared to respective controls. *RFC3* expression was slightly increased at H_2_O_2_ 1 h and repair 3 h groups in CEFs, significantly highest at H_2_O_2_ 1 h group in EGK.X blastoderm cells, and a slight increase at H_2_O_2_ 1 h group in PGCs. *DNTT* expression was slightly increased from H_2_O_2_ 1 h to repair 6 h groups in CEFs, slightly increased at H_2_O_2_ 1 h and repair 3 h groups in EGK.X blastoderm cells, and highest at repair 6 h group in PGCs. *FAM175A* expression was slightly increased from H_2_O_2_ 1 h to repair 6 h groups in CEFs, slightly higher at H_2_O_2_ 1 h group but significantly higher in a decreasing manner from repair 3 h to repair 12 h groups in EGK.X blastoderm cells, and significantly highest at H_2_O_2_ 1 h group in PGCs.

In the results of apoptosis pathway genes (Fig. 8), *FAS* expression was significantly increased at H_2_O_2_ 1 h group; however, it showed a decreasing manner from repair 3 h to repair 12 h groups in CEFs. In contrast, *FAS* expression showed an increasing manner from H_2_O_2_ 1 h to repair 12 h groups in EGK.X blastoderm cells. In PGCs, *FAS* expression was significantly increasing at repair 6 h and repair 12 h groups. The expression of *BAK1* was not significantly different in CEFs at H_2_O_2_ 1 h to repair 6 h groups, though a slight increase at repair 12 h group. In contrast, *BAK1* showed a significantly increasing pattern in EGK.X blastoderm cells (from H_2_O_2_ 1 h to repair 12 h groups) and PGCs (from repair 3 h to repair 12 h groups). Lastly, the pluripotency regulating gene (*NANOG*) was either decreased or not detectable in all the experimental groups of CEFs, EGK.X blastoderm cells, and PGCs (Fig. 8).

## Discussion

Compared to the pluripotency-related and germ cell development-related studies, genome stability-related studies are deficient in the chicken blastoderms and PGCs. Therefore, we employed the transcriptome sequencing method to comprehensively analyze the expression patterns of different DNA repair pathway genes and apoptosis pathway genes, along with candidate pluripotency regulating genes, in the chicken blastoderms and PGCs. In the hierarchical cluster analysis, DNA repair pathway genes were exclusively identified in 22 clusters. In comparison, the apoptosis pathway genes were exclusively identified in 6 clusters, and pluripotency genes were exclusively identified in 11 clusters. This result indicates that several DNA repair pathway genes show unique expression patterns and actively maintain the genome stability of cells from early chicken embryos. We further noted that the cluster combinations containing DNA repair pathway genes were higher than those containing apoptosis pathway genes or pluripotency regulating genes. To repair DNA damage and avoid passing it to a progeny of cells, the cell is equipped with multiple repair pathways, which can act independently or often interact (crosstalk)^18^.

In the expression profiling of DNA repair pathway genes, most of the genes were showed stronger expression primarily in the blastoderms and then in PGCs compared to CEFs. In contrast, most of the apoptosis pathway genes were showed stronger expression in the CEFs than that of blastoderms and PGCs. These results clearly show that the blastoderms maintain very strong genome stability, which requires an efficient cellular DNA repair mechanism, thus ensuring the normal development of post-oviposited embryos without any mutations and chromosomal instability. Second, PGCs also maintain strong genome stability, thus ensuring the normal development of gametes (and next-generation) without any mutations and chromosomal instability. To our knowledge, there is no report on the expression of DNA repair pathway genes in the chicken blastoderms. Likewise, there is only one report on the expression of DNA repair (BER) pathway genes in the chicken (primordial) germ cells. In that study, several BER pathway genes were significantly upregulated in PGCs than CEF cell line (DF-1) by RT-qPCR analysis, and subsequent functional studies confirmed that the base editing activity in PGCs could be regulated by modulating the expression of *UNG*, an upstream gene of the BER pathway^35^. The results that the higher expression of apoptosis genes in the normal CEFs could be a potential reason for hampering the long-term culture of primary CEFs^12^. However, the spontaneously developed immortalized CEF cell line (DF-1) is able to culture long-term and available for various biological applications, including viral amplification and gene editing^36–39^. In the expression profiling of candidate pluripotency regulating genes, a limited number of genes, including *NANOG*, *Pou5f3*, and *SOX2*, were significantly upregulated in both blastoderms and PGCs than that of CEFs. These three genes are the core transcriptional network of the pluripotent stem cells and have essential roles in early embryonic development^40^. Studies in mouse (naive state of pluripotency) and human (primed state of pluripotency) reports that different signaling pathways (including the MAPK signaling, TGFβ signaling, and Wnt signaling) involve in activating the core transcriptional network^41–43^. After activation, the core transcriptional network regulates several downstream genes for the maintenance of pluripotency^40^. On the other hand, the core transcriptional network was also reported as important for the specification and development of PGCs in mammals. In this case, WNT3-β-catenin and BMP-SMAD signaling activates the core transcriptional network via a core regulatory network consisting of *PRDM1*, *PRDM14*, and *TFAP2C*^44,45^. Although fragmentary, the expression patterns of several above pluripotency regulating signaling genes and core transcriptional/regulatory network genes were reported during the dynamic development of chicken early embryos and germ cells^16,31–34,46–48^.

The in vitro cultured CEFs, EGK.X blastoderm cells, and PGCs were treated with 1 mM H_2_O_2_ for 1 h and then incubated in fresh media without H_2_O_2_ for 3–12 h to examine the cell status after inducing DNA damage. The cells from all groups were subjected to TUNEL assay (to detect apoptotic cells with DNA strand breaks), γ-H2A.X immunocytochemistry (to detect cells undergoing DNA repair), and WST-1 assay (to detect the relative proliferation rate of cells). A dose and duration-dependent induction of DNA damage by H_2_O_2_ treatment has been reported in several cells. Particularly, treatment with 1 mM H_2_O_2_ for 1 h or longer period significantly affects the cells as evidenced by reduced cell number due to DNA damage-induced apoptotic cell death and increased expression of γ-H2A.X in cells undergoing DNA repair^49–51^. Phosphorylation of H2AX plays a critical role in DDR and is required for the assembly of DNA repair proteins at the sites of DNA damage. Several phosphoinositide 3-kinase-related protein kinases, such as ataxia teleangiectasia mutated (ATM), ATM and Rad3-related (ATR), or DNA-dependent protein kinase (DNA-PK), are considered as the major physiological mediator of H2AX phosphorylation^22,52^. Analysis of phosphorylated H2AX (γ-H2A.X) expression is widely used to detect the genotoxic effect in cells exposed to various toxic substances and in cells where genome stability-related genes are affected^52–54^. According to our results of the TUNEL assay, DNA damage was successfully induced in CEFs, blastoderm cells, and PGCs after treatment with H_2_O_2_. When all the cells were allowed to undergo DNA repair, however, a considerable number of CEFs were positive to γ-H2A.X compared to blastoderm cells and PGCs. Also, the WST-1 assay shows a significant increase in the proliferation rate of CEFs than that of blastoderm cells and PGCs. These results indicated that the EGK.X blastoderm cells and PGCs were more likely to undergo apoptotic cell death when their DNA was damaged.

To examine the expression status of CEFs, EGK.X blastoderm cells, and PGCs at DNA damage-induced condition, candidate DNA repair, apoptosis, and pluripotency genes were amplified in the cDNA of cells from H_2_O_2_ 1 h to repair 12 h groups. The results collectively indicate a time- and cell-specific upregulation of DNA repair pathway genes (*NEIL2*, *RFC3*, *DNTT*, and *FAM175A*) in response to H_2_O_2_ treatment. Particularly, the HR pathway gene *FAM175A* (in blastoderm cells and PGCs) and the NHEJ pathway gene *DNTT* (in PGCs) were predominantly higher in the target samples compared to the reference sample. Several factors could contribute to the cell’s choice of preferred DNA repair machinery when the cell detects DNA damage. The cell determines the appropriate repair pathway based on the source of the damage, nature of the damage, availability of a potential repair template, cell cycle phase, and the expression of key genes^18,19,22,55^. Specifically: BER corrects those forms of oxidative, deamination, alkylation, chlorination, and abasic single base damage; NER removes bulky lesions such as cyclobutane pyrimidine dimers and pyrimidine-(6–4)-pyrimidone photoproducts, or damage from chemotherapeutic agents; MMR corrects base mismatches that arise during replication^18,19^. NHEJ occurs throughout the cell cycle, especially in the G1 phase. This pathway does minimal processing around the break site and ligation of the ends; therefore, it is more frequently error-prone. HR occurs mainly in S and G2 phases. This pathway utilizes the sister chromatid or homologous chromosome; therefore, it is an accurate repair pathway^22,55^.

In our results, both the extrinsic (*FAS*) and intrinsic (*BAK1*) apoptosis pathway genes were showed a significantly increasing manner. In contrast, the pluripotency gene (*NANOG*) was completely decreased in blastoderm cells and PGCs in response to H_2_O_2_ treatment. It was reported in mammals and lower vertebrates that the mutation frequency is much lower in ESCs and germ cells than embryonic fibroblasts and other somatic cells when the DNA damage was induced, and the decision-making pathways that the ESCs and germ cells select are mostly different from somatic cells^56–58^. In response to DNA damage, somatic cells are more likely to undergo cell cycle arrest and attempt DNA repair, including the predominant utilization of the error-prone NHEJ pathway. In contrast, pluripotent stem cells and germ cells are subject to apoptosis, and when DNA repair is attempted, these cells utilize the error-free HR as a predominant repair pathway^58^. Chicken blastoderm cells (which are mostly pluripotent cells) and PGCs share some features, including the expression of pluripotency genes^46^. Therefore, the observed decision-making pathways in mammalian pluripotent stem cells and germ cells in response to DNA damage could also be observed in chicken blastoderm cells and PGCs.

## Conclusions

The findings of this study collectively indicate that the chicken EGK.X blastoderms that contain mostly undifferentiated pluripotent cells, and the PGCs that differentiate into male and female germ cells, show WTS-based higher expression of DNA repair pathway genes and the lower expression of apoptosis pathway genes when compared to CEFs. Among the pluripotency regulating genes, the critical core transcriptional network genes were strikingly higher in EGK.X blastoderms and PGCs than CEFs. When the DNA damage was induced, EGK.X blastoderm cells and PGCs were more likely to undergo apoptotic cell death along with the loss of pluripotency and less likely to undergo DNA repair. These properties of EGK.X blastoderms and PGCs should be necessary to preserve genome stability and avoid any mutations and chromosomal instability during the post-ovipositional early embryo development and germ cells development, respectively.

## Materials and methods

### Experimental animals and animal care

The care and experimental use of White Leghorn (WL) chickens was approved by the Institute of Laboratory Animal Resources, Seoul National University, Korea. Chickens were maintained according to a standard management program at the University Animal Farm, Seoul National University. All procedures, including chicken maintenance, reproduction, and sample collection, were governed by standard operating protocols according to a standard management program at the University Animal Farm, Seoul National University and the Animal Genetic Engineering Laboratory at Seoul National University.

### Collection of CEFs, EGK.X blastoderms, and PGCs

We collected three test samples, including CEFs, EGK.X blastoderms, and PGCs, for this study. The freshly laid WL eggs were incubated with intermittent rocking at 37.5 °C under 60–70% relative humidity until the day of sample collection. For the collection of CEFs, all internal organs and limbs were removed from the embryos at Hamburger and Hamilton (HH)^2^ stage 28/embryonic day 6 (N = 5 for each replication). The remaining embryonic body was then dissociated using 0.05% trypsin-EDTA (Gibco-Thermo Fisher Scientific, Inc., Waltham, MA, USA) in Hanks’ Balanced Salt Solution (HBSS; Gibco) at 37 °C for 10 min. After adding 10% fetal bovine serum (FBS; HyClone Laboratories, Logan, UT, USA) for inactivation of trypsin-EDTA and briefly centrifuging at 1200 rpm for 5 min, cells were cultured in Dulbecco’s modified Eagle’s medium (DMEM/High Glucose; HyClone) supplemented with 10% FBS and 1% antibiotic–antimycotic (Gibco) at 37 °C in an atmosphere with 5% CO_2_ and 60–70% relative humidity^59^. The adherent CEFs were subcultured at 3–4 days intervals via trypsin–EDTA treatment until passage 2.

EGK.X blastoderms^1^ were collected (N = 10 for each replication) from freshly laid eggs. After opening the egg, a small piece of square filter paper (Whatman, Maidstone, UK) with a hole in the center was placed over the germinal disc. After cutting around the filter paper containing the embryo, it was gently turned over and transferred to phosphate-buffered saline (PBS) to remove the yolk and vitelline membrane and allow embryo collection^60^. The collected EGK.X blastoderms were mechanically dissociated into single cells in PBS. For the collection of PGCs, gonads at HH stage 28 were dissociated using 0.05% trypsin–EDTA in HBSS. After adding 10% FBS for inactivation of trypsin–EDTA and briefly centrifuging at 1200 rpm for 5 min, cells were incubated for 20 min with anti-stage specific embryonic antigen (SSEA-1; Santa Cruz Biotechnology, Santa Cruz, CA, USA). The cells were washed with 1 mL of magnetic-activated cell-sorter (MACS) buffer (0.5% BSA and 2 mM EDTA), and the supernatant was completely removed by centrifugation. The pellet was mixed with 100 μL MACS buffer supplemented with 20 μL rat anti-mouse IgM microbeads for 15 min at 4 °C. The cells were washed by the addition of 500 μL MACS buffer and loaded into a MACS column (Miltenyi Biotec GmbH, Bergisch Gladbach, Germany) for the collection of MACS^+^ PGCs^61^. Approximately 6.3 × 10^5^, 7.8 × 10^5^, and 1.0 × 10^6^ PGCs were collected from 484, 658, and 684 gonads, respectively, for each replication. In addition, approximately 5000 MACS^+^ and MACS^-^ cells from each replication were immunostained with SSEA-1 to further confirm the MACS^+^ cells are PGCs.

### Generation and preprocessing of WTS data from CEFs, EGK.X blastoderms, and PGCs

Total RNA was isolated from CEFs, EGK.X blastoderms, and PGCs using Trizol reagent (Invitrogen, Carlsbad, CA, USA). The quality and quantity of the extracted total RNA were determined using the DropSense96 system (Trinean, Gentbrugge, Belgium), Ribogreen (Invitrogen), and the Agilent 2100 Bioanalyzer (Agilent Technologies, Santa Clara, CA, USA). Then, total RNA was used to construct cDNA libraries using the TruSeq Stranded Total RNA Sample Preparation kit with Ribo-Zero Gold (Illumina, San Diego, CA, USA). The resulting libraries were subjected to whole-transcriptome sequencing (WTS) analysis using the Illumina Nextseq 500 platform to produce 150 bp paired-end reads. Three biological replications were made for each sample, and a total of 9 raw-sequencing data are publicly available in the NCBI GEO database (GSE174603). To generate clean reads, we trimmed adapter sequences and removed low-quality reads from the raw paired-end sequenced files using Trimmomatic v.0.39^62^. The quality of the clean reads was quality-checked (QC) whether read lengths were longer than 75 bp and Phred scores were higher than 30 using FastQC v.0.11.9 (https://www.bioinformatics.babraham.ac.uk/projects/fastqc/).

### Alignment, quantification, and statistical analysis of reads

We mapped the clean reads to the galGal6a reference genome (GRCg6a) through HISAT2 v.2.2.0^63^ and converted the sequence alignment/map files (.SAM files) into binary alignment/map files (.BAM files) using SAMtools v.1.10^64^. Then, to quantify gene expression, we used HTSeq-count^65^ and counted the number of reads mapped to Ensembl genomic GTF of galGal6a release 101^66^. To normalize the read counts, trimmed mean of M values (TMM) were calculated and the dispersion parameters of samples were estimated^67^.

Differentially expressed genes (DEGs) between the samples were detected from negative binomial-based generalized linear models (GLM). In total, we performed three statistical tests, CEFs versus EGK.X blastoderms, CEFs versus PGCs, and EGK.X blastoderms versus PGCs. We used the ‘edgeR’ package implemented in R for the normalization and statistical testing^68^. Likelihood ratio tests were performed to obtain the *P*-values, and false discovery rates (FDR) were calculated from *P* values for multiple testing corrections. Genes of which FDR* P* < 0.05 were considered to be significant, while log_2_ *fold change* (logFC) > 1 were considered as upregulated and logFC < − 1 were considered as downregulated. Genes that fall between these logFC were considered unregulated^47^.

For visualization, we used R packages ‘ggplot2’ and ‘ComplexHeatmap’ to make a multidimensional scaling plot (MDS plot) and heatmaps from log_2_ *TMM* values. Also, we made volcano plots showing FDR and logFC with R package ‘EnhancedVolcano’ and Venn diagrams of alignment results with R package ‘VennDiagram’.

### WTS-based analysis of DNA repair pathway, apoptosis pathway, and pluripotency regulating genes

The organism (chicken)-specific DNA repair, including BER (gga03410), NER (gga03420), MMR (gga03430), NHEJ (gga03450), and HR (gga03440), pathway genes were primarily identified using the Kyoto Encyclopedia of Genes and Genomes (KEGG) pathway maps^69^. Similarly, the chicken-specific extrinsic and intrinsic apoptosis (gga04210) pathway genes were identified using the KEGG pathway maps^69^. The retrieved gene lists were updated according to the current genome versions of chickens (galGal6a) in the Ensembl database^66^, and also, the corresponding gene IDs and chromosome positions were collected. The identified genes of each DNA repair pathway and apoptosis pathway were again subjected to the KEGG database to understand their involvement in other pathways. The chicken homologs of signaling pathway genes regulating pluripotency of stem cells in human/mouse were identified using the KEGG pathway map (hsa04550)^69^ and Ensembl database^66^.

Hierarchical clustering was performed to examine the relationship between the DNA repair pathway, apoptosis pathway, and pluripotency regulating genes expression. We used 16,317 protein-coding genes whose expression is not zero and categorized them into DNA repair pathway, apoptosis pathway, pluripotency regulating, or other genes. Then, we used log_2_ *TMM* values to calculate the distances and the Ward linkage method to make clusters. Built-in functions of R software ‘hclust’ and ‘cutree’ were used to make 100 clusters. We summarized each cluster as the number of genes in the four categories is included. The quantification of gene expression, differential expression, and data visualization of DNA repair pathway, apoptosis pathway, and pluripotency regulating genes were the same as described above. In addition, violin plots using R package ‘ggplot2’ were prepared with the log_2_ *TMM* values to visualize the overall expression of different pathway genes. Furthermore, we performed gene set enrichment analysis (GSEA) using GSEA v.4.1.0 to find out which pathways are significantly enriched^70^. The gene lists used for GSEA were pre-ranked according to their logFC values. The gene sets to analyze were defined as the genes included in KEGG pathways related to DNA repair, apoptosis, and pluripotency.

### Culture of CEFs, EGK.X blastoderm cells, and PGCs

CEFs were cultured in DMEM/High Glucose supplemented with 10% FBS and 1% antibiotic–antimycotic at 37 °C in an atmosphere with 5% CO_2_ and 60–70% relative humidity until passage 2 as described above.

EGK.X blastoderms were dissociated using 0.05% trypsin–EDTA in HBSS at 37 °C for 10 min. After adding 10% FBS for inactivation of trypsin–EDTA and briefly centrifuging at 850 rpm for 5 min, cells were washed twice in PBS. Blastoderm cells were cultured as reported early with minor modifications in N2B27/2i medium containing DMEM/F-12 (Gibco), Neurobasal (Gibco), 55 mM β-mercaptoethanol (Gibco), 200 mM l-glutamine (Gibco), N2-Supplement (100×, Gibco), and B27 supplement-vitamin A (50×, Gibco). The medium was supplemented with two inhibitors: 3 μM CHIR99021 and 1 μM PD0325901 (Stemgent, San Diego, CA, USA)^71,72^. The adherent blastoderm cells were cultured for up to 3 days at 37 °C in an atmosphere with 5% CO_2_ and 60–70% relative humidity, without subculture, and subjected for further experiments.

PGCs were cultured as reported early in knockout DMEM (Gibco) supplemented with 20% FBS, 2% chicken serum (Millipore Sigma, Burlington, MA, USA), 1 × nucleosides (Millipore Sigma), 1 × glutamax (Gibco), 1 × non-essential amino acids (Gibco), β-mercaptoethanol (Gibco), 1 mM sodium pyruvate (Gibco), 1 × antibiotic–antimycotic (Gibco), and 10 ng/mL human basic fibroblast growth factor (bFGF; Millipore Sigma) at 37 °C in an atmosphere with 5% CO_2_ and 60–70% relative humidity^73^. The suspension PGCs were subcultured at 4–5 days intervals via gentle pipetting, without any enzyme treatment, and centrifugation at 1200 rpm.

### Treatment of CEFs, EGK.X blastoderm cells, and PGCs with H_2_O_2_

Approximately 1 × 10^5^ CEFs, EGK.X blastoderm cells, or PGCs were seeded per well in 12-well plates. The adherent CEFs and EGK.X blastoderm cells were subjected to the treatment on day 3 of culture (when they reached 70–80% confluence), while the suspension PGCs were subjected to the treatment on day 1 of culture. The first group was left as control. In the second group, cells were treated with 1 mM hydrogen peroxide (H_2_O_2_; Sigma-Aldrich, St. Louis, MO, USA) for 1 h (1 h). In the third, fourth, and fifth groups, cells were first treated with 1 mM H_2_O_2_ for 1 h, and then incubated in fresh media without H_2_O_2_ for 3 h, 6 h, and 12 h, respectively. The treated cells were harvested at the end of each experimental time point by centrifugation and washing with PBS and subjected for further analysis. Control cells were harvested together with the time of H_2_O_2_ for 1 h. Each treatment for further analysis was performed in triplicate.

### TUNEL assay

The control and H_2_O_2_ treated CEFs, EGK.X blastoderm cells, and PGCs were fixed with 4% paraformaldehyde (Sigma-Aldrich) for 15 min and permeabilized with 0.1% Triton X-100 (Sigma-Aldrich) for 10 min. Apoptotic cells with DNA strand breaks were identified using an In Situ Cell Death Detection Kit, TMR-red (Roche Diagnostics GmbH, Mannheim, Germany). Cells were counterstained with 4′,6-diamidino-2-phenylindole (DAPI; Vector Laboratories, Inc., Burlingame, CA, USA) and analyzed under a fluorescence microscope (Eclipse Ti; Nikon, Tokyo, Japan).

### Immunocytochemistry

The control and H_2_O_2_ treated CEFs, EGK.X blastoderm cells, and PGCs were fixed with 4% paraformaldehyde for 15 min and permeabilized with 0.1% Triton X-100 for 10 min. After blocking the non-specific binding with 1% bovine serum albumin (Sigma-Aldrich) for 1 h, cells were incubated with mouse anti-γ-H2A.X antibody (phospho S139) (Abcam, Cambridge, UK) at a 1:200 dilution in blocking buffer for overnight at 4 °C to detect the cells undergoing DNA repair. Cells were then incubated with Alexa Fluor 488-conjugated goat anti-mouse IgG secondary antibody (Abcam) at a 1:500 dilution in the blocking buffer for 1 h. Cells were counterstained with DAPI and analyzed under a fluorescence microscope (Eclipse Ti).

### WST-1 assay

The control and H_2_O_2_ treated CEFs, EGK.X blastoderm cells, and PGCs were harvested by centrifugation and washing with PBS. Then, the cells were resuspended in the respective cell culture media containing WST-1 premix (Takara, Tokyo, Japan) at a 1:10 dilution. The complex was aliquated equally to the wells of 96-well plates and incubated at 37 °C, 5% CO_2_ for 2 h. The absorbance of the samples against a blank was measured using a VersaMax microplate reader (Molecular Devices, San Jose, CA, USA). Significant differences between the respective control and treated samples were determined by Student’s *t* test using the GraphPad Prism software (San Diego, CA, USA). Statistical significance was ranked as *P < 0.05, **P < 0.01, ***P < 0.001, or ****P < 0.0001.

### RT-qPCR analysis

First, we performed RT-qPCR analysis to further confirm the WTS results of selected DNA repair and apoptosis pathway genes in the CEFs, EGK.X blastoderms, and PGCs. Second, we performed RT-qPCR analysis to examine the selected DNA repair pathway, apoptosis pathway, and pluripotency regulating genes in control and H_2_O_2_ treated CEFs, EGK.X blastoderm cells, and PGCs. According to the manufacturer's instructions, the total RNA of the test samples was isolated using the Trizol reagent, and the RNA quantity was determined by Nanodrop2000 (Thermo Fisher Scientific). cDNA was synthesized from the RNA using the Superscript III First-Strand Synthesis System (Invitrogen). The qPCR reaction mixture contained 2 μL of PCR buffer, 0.4 μL of 10 mM dNTP mixture, 10 pmol each of gene-specific forward and reverse primers, 1 μL of 20 × EvaGreen (Biotium, Hayward, CA, USA), 0.2 μL of Taq DNA polymerase, and 2 μL of cDNA to a final volume of 20 μL. The qPCR was performed using a StepOnePlus real-time PCR system (Applied Biosystems, Foster City, CA, USA) with the thermal condition contained an initial incubation at 95 °C for 5 min, followed by 40 cycles at 95 °C for 30 s, 60 °C for 30 s, and 72 °C for 30 s. The reaction was ended after final incubation at the system’s dissociation temperature. The relative quantification of the gene expression was normalized with the chicken *GAPDH* and a reference sample (CEFs for the first qPCR; respective control for the second qPCR), and analyzed by the 2^−ΔΔCt^ method. Significant differences between the reference and other samples were determined by Student’s *t* test using the GraphPad Prism software. Statistical significance was ranked as *P < 0.05, **P < 0.01, ***P < 0.001, or ****P < 0.0001.

### Ethics statement

All experimental procedures and care of chickens was approved by the Institute of Laboratory Animal Resources, Seoul National University, and all methods were carried out in accordance with ARRIVE (Animal Research: Reporting of In Vivo Experiments) guidelines and approved by the Institutional Animal Care and Use Committee (IACUC, SNU‐190401‐1-1 and SNU‐200519-2) of Seoul National University, Korea.

### Approval for animal experiments

All experimental procedures and care of chickens was approved by the Institute of Laboratory Animal Resources, Seoul National University. All methods were carried out in accordance with the guidelines and regulations of the Institutional Animal Care and Use Committee of Seoul National University (IACUC, SNU‐190401‐1-1 and SNU‐200519-2). All procedures, including chicken maintenance, reproduction, and sample collection, were governed by standard operating protocols according to a standard management program at the University Animal Farm, Seoul National University and the Animal Genetic Engineering Laboratory at Seoul National University.

## Supplementary Information

Supplementary Information 1.

Supplementary Information 2.

## Data Availability

Raw-sequencing data of the current study are publicly available in the NCBI GEO database (GSE174603). Other datasets generated during and/or analyzed during the current study are available from the corresponding author on reasonable request.
