# Supplementary material for: Chicken blastoderms and primordial germ cells possess a higher expression of DNA repair genes and lower expression of apoptosis genes to preserve their genome stability
Source: Sci Rep. 2022 Jan 7;12:49. doi: 10.1038/s41598-021-04417-y (PMC8741993; doi:10.1038/s41598-021-04417-y)
Supplement: Supplementary file 1 — Supplementary Information 1. [file 41598_2021_4417_MOESM1_ESM.pdf]

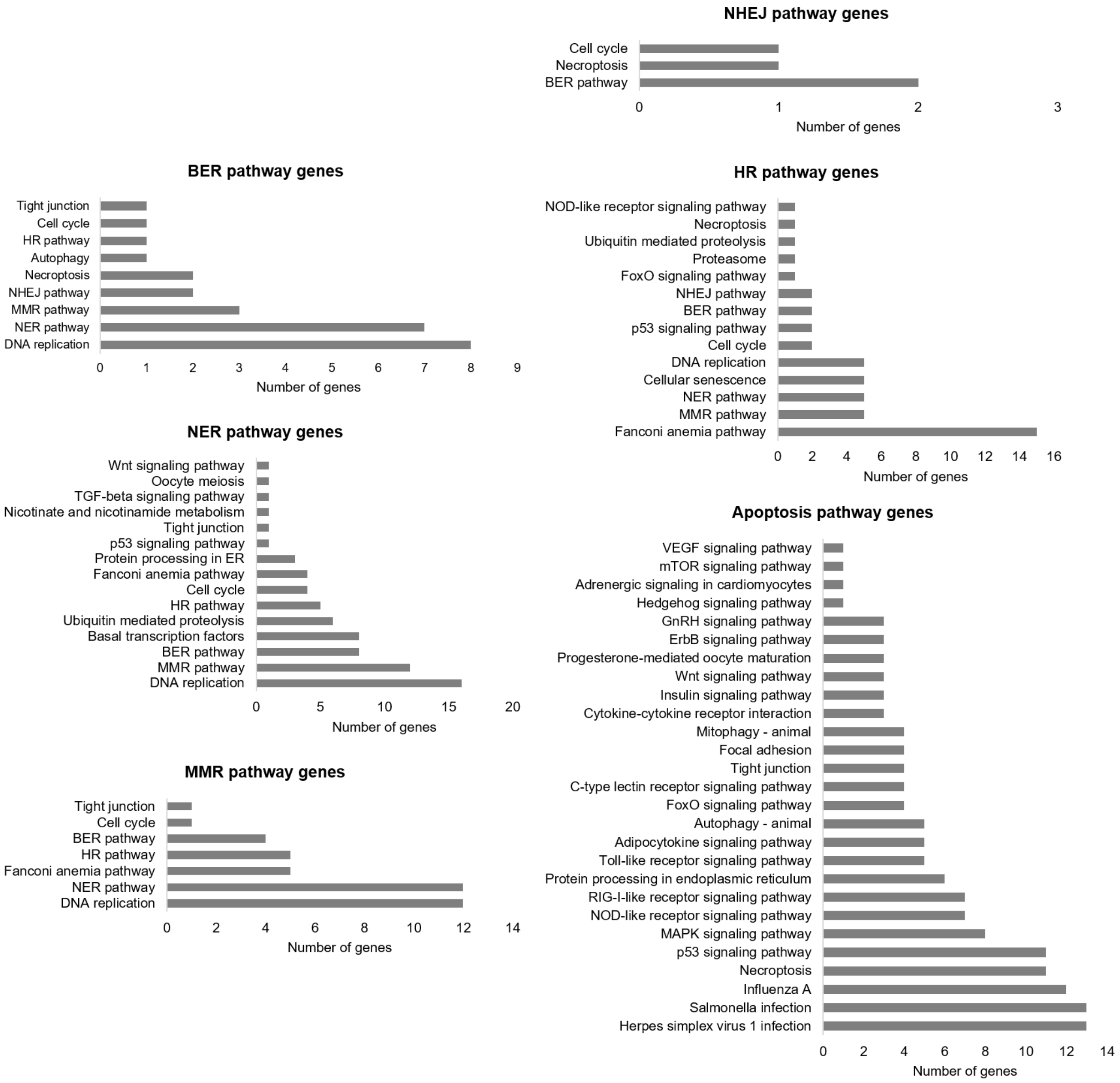

Supplementary Figure S1. Involvement of BER, NER, MMR, NHEJ, HR, and apoptosis pathway genes in other pathways.

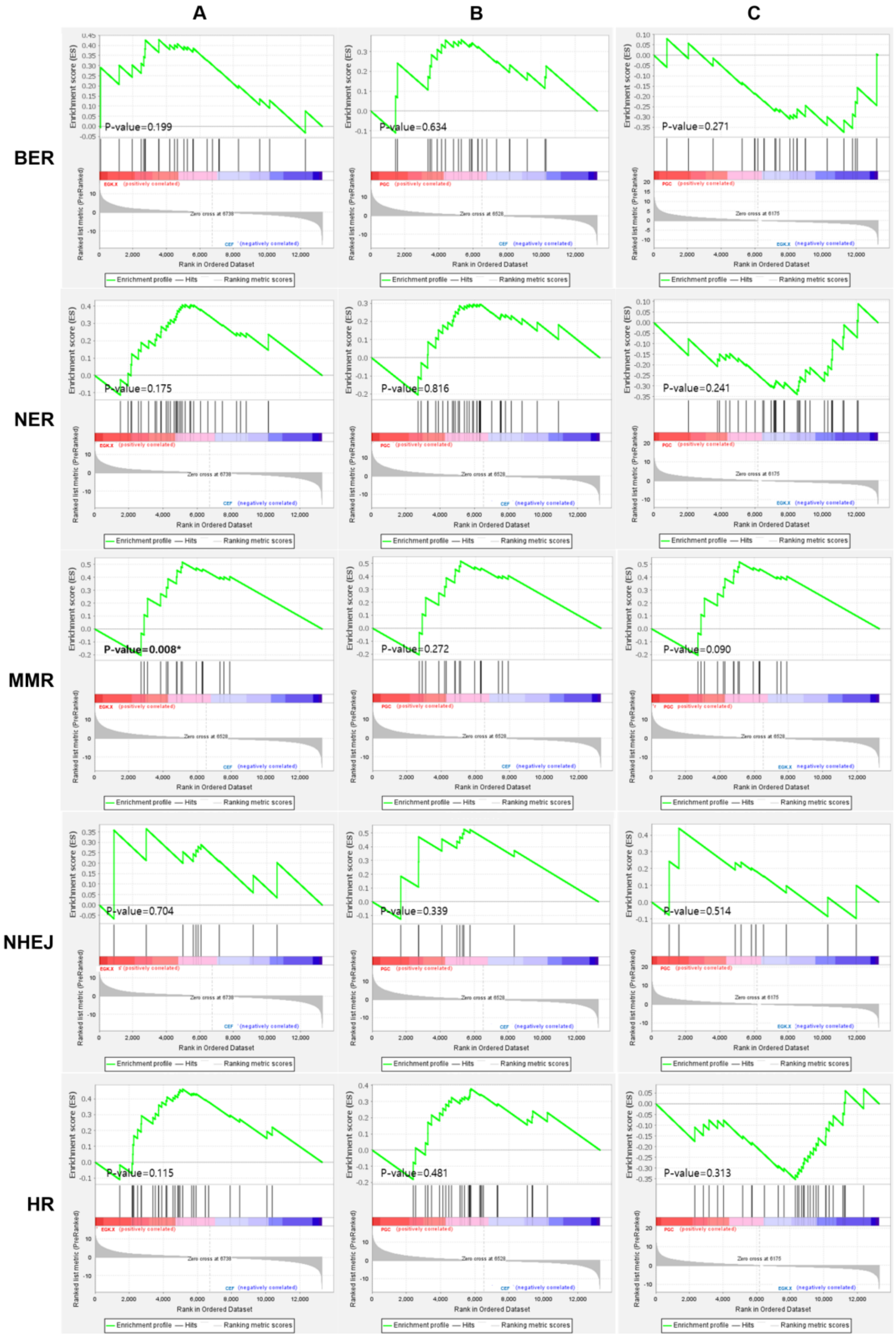

Supplementary Figure S2. GSEA of DNA repair (BER, NER, MMR, NHEJ, and HR) pathway at CEFs versus EGK.X blastoderms (**A**), CEFs versus PGCs (**B**), and EGK.X blastoderms versus PGCs (**C**) conditions.

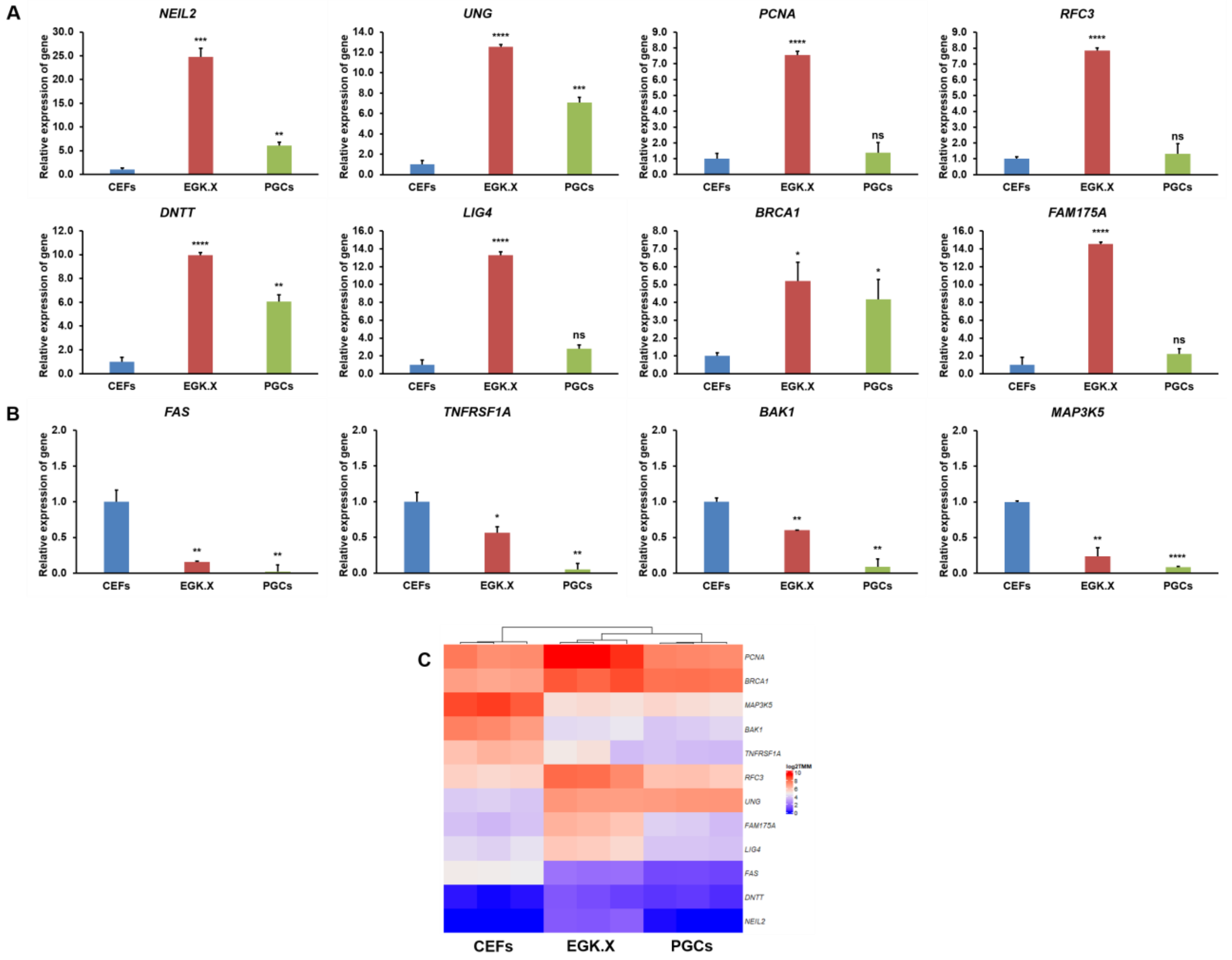

Supplementary Figure S3. RT-qPCR validation of DNA repair and apoptosis pathway genes. **A**, candidate genes from the BER pathway (*NEIL2* and *UNG*), NER/MMR pathway (*PCNA* and *RFC3*), NHEJ pathway (*DNTT* and *LIG4*), and HR pathway (*BRCA1* and *FAM175A*). **B**, candidate genes from the extrinsic (*FAS* and *TNFRSF1A*), intrinsic (*BAK1*), and FAS-JNK (*MAP3K5*) mediated apoptosis pathway. Genes were amplified with their specific qPCR primers. The relative expression of genes was normalized with the chicken *GAPDH* and a reference sample (CEFs), and analyzed by the $2^{-\Delta\Delta Ct}$ method. Significant differences between the CEFs and other test samples were determined by Student's *t*-test. Statistical significance was ranked as * $P < 0.05$, ** $P < 0.01$, *** $P < 0.001$, or **** $P < 0.0001$. ns: non-significant. **C**, Heatmaps using log2 *TMM* values of the normalized read counts of genes shown in **A** and **B** were prepared separately for better visualization.
